# Neonicotinoid-induced impairment of odour coding in the honeybee

**DOI:** 10.1038/srep38110

**Published:** 2016-12-01

**Authors:** Mara Andrione, Giorgio Vallortigara, Renzo Antolini, Albrecht Haase

**Affiliations:** 1 Center for Mind/Brain Sciences, University of Trento, Rovereto, Italy; 2 Department of Physics, University of Trento, Trento, Italy

## Abstract

Exposure to neonicotinoid pesticides is considered one of the possible causes of honeybee (*Apis mellifera*) population decline. At sublethal doses, these chemicals have been shown to negatively affect a number of behaviours, including performance of olfactory learning and memory, due to their interference with acetylcholine signalling in the mushroom bodies. Here we provide evidence that neonicotinoids can affect odour coding upstream of the mushroom bodies, in the first odour processing centres of the honeybee brain, *i.e.* the antennal lobes (ALs). In particular, we investigated the effects of imidacloprid, the most common neonicotinoid, in the AL glomeruli via *in vivo* two-photon calcium imaging combined with pulsed odour stimulation. Following acute imidacloprid treatment, odour-evoked calcium response amplitude in single glomeruli decreases, and at the network level the representations of different odours are no longer separated. This demonstrates that, under neonicotinoid influence, olfactory information might reach the mushroom bodies in a form that is already incorrect. Thus, some of the impairments in olfactory learning and memory caused by neonicotinoids could, in fact, arise from the disruption in odor coding and olfactory discrimination ability of the honey bees.

The effects of sublethal doses of neonicotinoid pesticides on pollinating insects have been extensively investigated^1^. Neonicotinoids, whose application has grown considerably over the last 20 years, have been reported by many studies to play a role in the observed decline of honeybee colonies^2^ representing a serious environmental and economic concern^3^,^4^. Various modifications in honeybee behaviour, which seem to be associated with cognitive impairment, have been described. General motion^5^,^6^, waggle dancing^7^, homing flights^8^,^9^,^10^, and olfactory learning and memory^11^,^12^,^13^,^14^ were shown to be disrupted by chronic and acute treatment with various neonicotinoids at doses comparable to those experienced by animals in the field.

The action of neonicotinoid pesticides on the insect brain and their influence on behaviour are caused by an interference with the acetylcholine pathway. All commercially available neonicotinoids are, in fact, agonists of the insect’s nicotinic acetylcholine receptors (nAChRs), perhaps the most widespread receptors in the insect central nervous system^15^. Nicotinic receptors are, among others, pivotal to the flow of olfactory information throughout the brain.

In the honeybee, odorants activate olfactory receptors (ORs) expressed at the surface of olfactory receptor neurons (ORNs) hosted in the antennae. ORNs forward the information to the antennal lobe (AL) glomeruli. There, ORN axons synapse with dendrites of projection neurons (PNs)–the second order neurons of the olfactory pathway–and with local neurons (LNs). Glomeruli receive direct input from a single OR family, and are interconnected by the LNs, which are mainly inhibitory. The olfactory information is then transferred by the PNs through five main tracts (the medial- and lateral-antenno-protocerebral tracts, m-APT and l-APT, and three mediolateral-antenno-protocerebral tracts, ml-APTs) to higher brain areas, such as the calyxes of the mushroom bodies (MBs) and the lateral horns (LHs). There, it is integrated with information from other sensory modalities and with previous memories, providing context^16^.

nAChRs are present both at the synaptic interface between the ORNs and the AL cells^17^ and between the PNs belonging to the m-APT^17^,^18^ and the mushroom bodies’ Kenyon cells (KCs).

Imidacloprid, the most widely used neonicotinoid pesticide, is a partial ligand of the insect nAChR, with a reported EC_50_ between 0.53 μM^19^ and 25 μM^20^ for cultured KCs at different developmental stages, and between 0.83 μM^21^ and 3.4 μM^22^ for cultured adult AL neurons. Imidacloprid has been shown to impair olfactory learning and memory in a number of studies^11^,^13^,^14^,^23^,^24^,^25^. As revealed by *in vivo* electrophysiological recordings, imidacloprid application produces a tonic inward current in Kenyon cells of the MBs, making them unresponsive to acetylcholine. This current is blocked by d-tubocurarine, and can therefore be ascribed to the sustained activation of KC nAChRs^26^. This result confirmed and extended *in vitro* evidence previously collected from dissociated cells^19^,^20^.

The above-described inactivation of Kenyon cells is thought to be the main cause of the impairments observed in bee navigation and in learning and memory. Indeed, these cells represent a major site of convergence for information across different sensory modalities and play an essential role in establishing and retrieving memories^27^. However, forming odour-specific memories requires correct stimulus encoding. Antennal lobes (ALs) are the brain areas responsible for this processing stage.

Nicotinic receptor expression in the ALs has been described before^17^. Dissociated cells from the AL have been shown to be affected by imidacloprid, although the identity of the responsive cells was not specified^21^,^22^. The experiments reported here aimed to elucidate the effects of acute imidacloprid treatment on signal processing in the AL and on the resulting response code in the PNs, the AL’s output units. These effects could not be predicted from results of *in vitro* studies, due to the cell type discrimination problem that was mentioned before but also, in particular, due to the network structure of the AL, which involves complex interactions among the single components.

To visualize the odour response maps, an *in vivo* functional imaging approach was used^28^. This technique has the advantage of preserving the multidimensionality of the odour code, since it allows simultaneous recordings from several distinct glomeruli. We monitored intracellular calcium changes within PNs over many cycles of stimulus repetitions with different odorant molecules and measured the effects of an acute application of imidacloprid on these activity patterns.

## Results

Staining PNs with fura 2-dextran allowed *in vivo* functional imaging of the AL glomerular responses upon stimulation with different odorants (1-hexanol, 1-octanol, acetophenone, and benzaldehyde).

Experiments were designed to allow continuous imaging of odour-stimulated activity from the same AL area (a single focal plane allowed access to 14 to 21 glomeruli depending on the bee) before, during, and after treatment of the brain with an imidacloprid solution. This was achieved via controlled perfusion of the brain with a buffer solution, to which substances could be added during well-defined periods, without creating interruptions or movement artefacts in the images.

**Figure 1:**
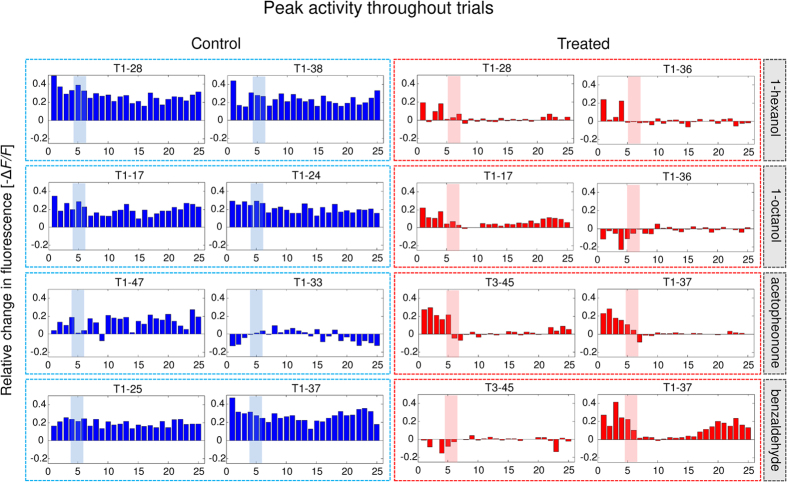
Example of two representative glomeruli of the odour response pattern relative to each odour in a control bee (left; blue) and a treated bee (right; red). Odour-evoked responses (−Δ*F/F*) are averaged over the 200–400 ms interval after stimulus onset, for each of the 25 stimuli repetitions (shown on the *x*-axis). Odours are reported on the right. Shadows: administration of Ringer’s solution (blue) or imidacloprid (red).

The administration of a 10 μM solution of imidacloprid for 1 min, followed by washing with physiological solution, resulted in significant reduction in the mean and peak odour-evoked calcium responses within single glomeruli (see Fig. 1 and Supplementary Fig. S1). A control experiment using single-dose administration under static conditions produced comparable results on the glomerular response at as little as one-tenth of that concentration (see Supplementary Fig. S2).

The onset of the effects on brain activity was slightly different among bees. This might be due to variations in the kinetics of perfusion and diffusion of the substance into the tissue. Around 1 min after the end of imidacloprid application, however, the effect on the odour-evoked responses was visible in all bees. A few minutes after imidacloprid administration, on the other hand, the response amplitude in some of the glomeruli recovered to initial values (Fig. 1 and Supplementary Fig. S1), though calcium transient dynamics could still be different. In one case, not all odour responses followed the same trend, and the recovery was odour-specific (Fig. 1, Glomerulus T1–37).

Due to the temporal variability mentioned above, and in order to compare effects across animals, we focused on three time windows, each encompassing three stimulus repetitions (≈1.5 min): before treatment, 1 min after the end of treatment (EOT), 8 min after EOT.

**Figure 2:**
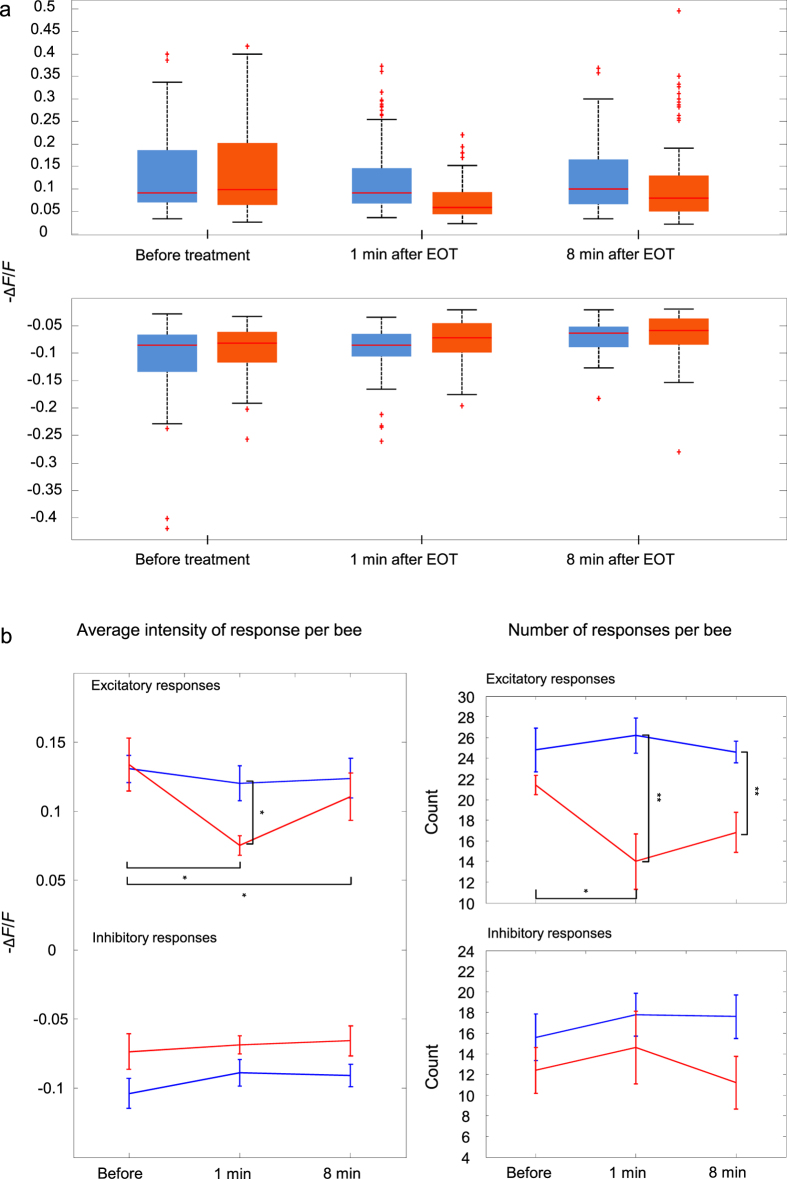
Odour-evoked glomerular responses were gathered and automatically classified in excitatory, inhibitory, or no response. This was done for responses before treatment (Before), 1 min after EOT (1 min) and 8 min after EOT (8 min). The intensity (average −Δ*F/F* between 200 and 400 ms after odour onset) distributions of excitatory (top) and inhibitory responses (bottom) are shown in (**a**), with data from control animals (*n* = 5) reported in blue and those from treated animals (*n* = 5) in red. Boxplots indicate median, quartiles, and outliers of the distributions. In (**b**), the average intensity (−Δ*F/F;* left) and the number (right) of both excitatory and inhibitory responses across bees are shown (control bees, *n* = 5, in blue, and treated bees, *n* = 5, in red). Error bars represent SEM. Average intensity of excitatory responses varied following treatment (two-way repeated measures ANOVA showed a significant effect of time *F*_2,16_ = 9.4, *p* = 0.0020 and group × time interaction, *F*_2,16_ = 4.7 *p* = 0.025). Number of excitatory responses were also reduced by the treatment (two-way repeated measures ANOVA showed a significant effect of group × time interaction, *F*_2,16_ = 3.9, *p* = 0.042, and group: *F*_1,8_ = 17, *p* = 0.003). Pairs of measurements were further compared via paired and unpaired *t*-tests, respectively (*p < 0.05, **p < 0.01).

First, we evaluated the distributions of “excitatory” and “inhibitory” odour-evoked responses (see Methods) in the two groups relative to the three moments (Fig. 2a). Shrinkage of the excitatory response distribution in treated animals following treatment (red, 1 min after EOT) suggested that these responses diminish in both intensity and number compared with those of control bees. We tested this hypothesis by comparing the number of excitatory/inhibitory responses and their average intensity in bees of each group (Fig. 2b).

Indeed, we found that both number and average intensity of excitatory responses were reduced in treated bees following treatment. The two-way repeated measures ANOVA on response number revealed significant effects of group: *F*_1,8_ = 17, *p* = 0.003 and group × time interaction: *F*_2,16_ = 3.9, *p* = 0.042, while time was not significant: *F*_2,16_ = 2.0, *p* = 0.16. The two-way repeated measures ANOVA on average intensity showed a significant effect of time: *F*_2,16_ = 9.4, *p* = 0.0020 and group × time interaction: *F*_2,16_ = 4.7 *p* = 0.025, but not group: *F*_1,8_ = 1.1, *p* = 0.33. These effects did not occur in inhibitory responses (two-way repeated measures ANOVA on number: time: *F*_2,16_ = 0.86, *p* = 0.44, group × time: *F*_2,16_ = 0.53, *p* = 0.60, group: *F*_1,8_ = 2.2, *p* = 0.18; two-way repeated measures ANOVA on intensity: time: *F*_2,16_ = 1.5398, *p* = 0.24; group × time: *F*_2,16_ = 0.3, p = 0.74, group: *F*_1,8_ = 4.5, *p* = 0.07).

Parallel to this analysis, we computed average delays in onsets of odour-evoked responses in the same three time windows. Odour-evoked responses from treated bees show a significant increase in onset delay at both time points after the treatment (see Supplementary Fig. S4). This is probably contributing to the incomplete recovery that emerges from the analysis of average responses (calculated between 200 and 400 ms after stimulus onset; see Supplementary Fig. S3).

The results on the individual odour-evoked responses led to the question of how the odour codes and odour discrimination would be affected by the treatment.

To answer this question, as a measure of distinguishability, Euclidean distances (EDs) between pairs of odour-response patterns were computed, and the dynamics of this measure over the course of the 25 stimulations around imidacloprid administration were studied. ED dynamics relative to an example control bee and a treated bee are shown in the Supplementary Fig. S5.

**Figure 3:**
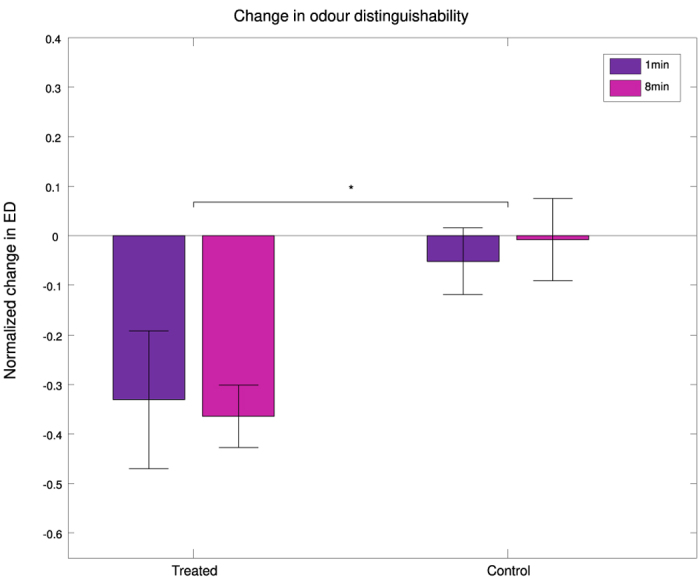
Normalized changes in Euclidean Distances over time (ED_after_ − ED_before_)/ED_before_ in treated and control bees. There is no significant effect of time on ED measurements, nor of time × group interaction. However, EDs are significantly reduced in the treated group at both time points after EOT (1 min: 1 min after EOT, shown in violet, and 8 min: 8 min after EOT, shown in fuchsia) with respect to the control group (*n* = 5 bees per group; two-way repeated measures ANOVA, group: *F*_1,8_ = 8.3, *p* = 0.020). Error bars represent SEM.

Again, to compare changes in EDs across bees, we focused on the three time windows described above (before treatment, 1 min after EOT, 8 min after EOT). In each bee, we extracted EDs relative to each period (see Supplementary Fig. S6). Then, we computed a measure of normalized change in ED: (ED_after_ − ED_before_)/ED_before_, for both time points after EOT. Normalized changes in ED were averaged across odour pairs, and compared across bees of both groups (Fig. 3).

The treated group showed a significant reduction in the diversity of the odour codes following treatment, with respect to the control, at both time points after treatment (two-way repeated measures ANOVA revealed a significant effect of group: *F*_1,8_ = 8.3. *p* = 0.020, but not of time: *F*_1,8_ = 0.28, *p* = 0.61, or group × time interaction: *F*_1,8_ = 0.29, *p* = 0.61).

**Figure 4:**
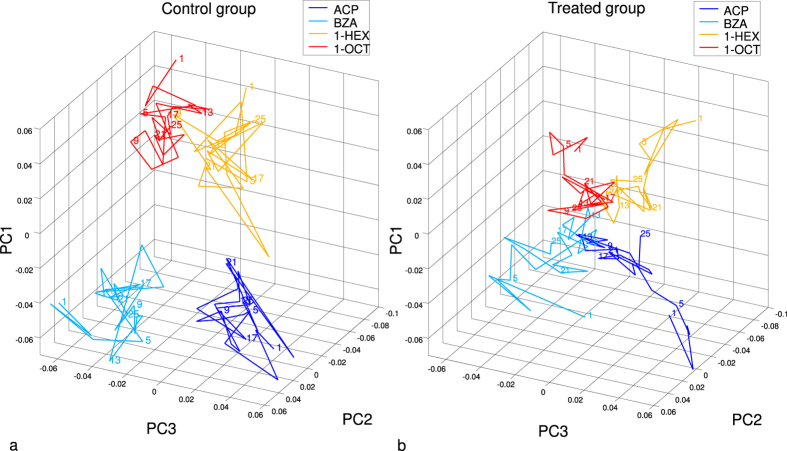
Average odour representation in time (*n* = 5 bees per group; ACP: acetophenone, shown in blue, BZA: benzaldehyde, in cyan, 1-HEX: 1-hexanol, in yellow, and 1-OCT: 1-octanol, in red) during 25 stimulus repetitions (repetitions are marked by numbers along the trajectories) in control (**a**) and treated (**b**) bees. Imidacloprid was administered on average between trials 4.6 and 6.6 to the treatment group (**b**), while, in the same window, the control group (**a**) was administered with Ringer’s solution from a second vial. Odour responses are shown in principal components (PCs) in order to reduce the coding space dimensionality. PCs and axes are identical for (**a**,**b**), allowing comparison of odour code separation.

To visualize the alteration of the response codes relative to the 4 odour stimuli during and after the treatment, we reduced the dimensionality of the coding space by a principal component analysis (PCA). Transforming the *n*-dimensional space of all recorded glomeruli in all bees (*n* = 172) we find that the first three principal components (PCs) explain all variance across mean activities. Subsequently, we averaged mean activities in PCs across bees within each group (*n* = 5). In this way we obtain for each odour a temporal sequence of the mean group response to the 25 repeated stimuli. The evolution of the mean responses to all odours for treatment and control group is shown in the three-dimensional PC space (Fig. 4).

The odour representations within the control group (Fig. 4a) were confined to a given area of the coding space, remaining stable over the course of 25 stimulation cycles. On the contrary, in the treated group (Fig. 4b) the representations of the 4 odours, which were well separated at the beginning, collapsed after treatment (administered on average between trials 4.6 and 6.6; see marker 5 and following, Fig. 4b) onto the origin of the coding space. A trend was noticeable in the final stimulation cycles (see *e.g.* marker 25, Fig. 4b) of some of the odours to separate again within the glomerular coding space. However, the overall distances between different odorants were still reduced compared to the initial condition.

## Discussion

Effects of imidacloprid on the dissociated antennal lobe cells have been previously described^21^,^22^. However, in those studies, it was not possible to determine the identity of the recorded cells, and it was therefore impossible to make any hypotheses on how this substance would affect the AL network when tested *in vivo.*

We have shown here that the odour-specific calcium transients evoked in the AL PNs are greatly reduced by imidacloprid application. This reduction is probably due to inactivation of PNs. Indeed, it has been demonstrated that, at least in the case of Kenyon cells, imidacloprid induces a tonic inward current, making the cells unable to respond to acetylcholine^26^. We assume the same happens in PNs, which would explain the vanishing of all odour-induced calcium response soon after treatment. The partial reversibility of the observed effect can be explained by a progressive detachment of imidacloprid molecules from their binding sites, supported by the sustained washing with the physiological solution. Additionally, a delayed recovery of the intracellular calcium concentration, initially raised by imidacloprid^29^, might contribute.

However, the observed reversibility was not complete, as it did not encompass all glomeruli. Moreover, in glomeruli in which the response amplitude was restored, sometimes the shape of the odour-evoked calcium transient was different compared to that of pre-treatment, as we showed in the case of response onsets, which were often delayed. All this prevented the odour code from being fully regained. In fact, the Euclidean distances–measures of distinguishability between odour pairs –, remained below initial values during the complete post-treatment period. So, even if the recovery on a single-glomerulus scale seemed to have saturated within ≈10 min after the EOT, some important part of the odour code was probably never regained. It has been previously suggested that different nAChRs might be expressed at different locations in AL^22^, and a possible explanation for our results is that these receptors show different sensitivity to imidacloprid. This is a point that will need to be further elucidated.

It seems reasonable to argue that PNs are not the only cell type responsible for the effects we observed. This is suggested *e.g.* by the response pattern of glomerulus T1–37 (Fig. 1 and Supplementary Fig. S3). Within the same glomerulus, two different odour-evoked responses were differently affected by the treatment, implying that the information pertaining to those two odorants is conveyed to these PNs via two distinct cellular pathways, *i.e.* as direct input from ORNs and via the LNs. Therefore, we hypothesise that this latter cell type (most probably also expressing nAChRs) might not be able to restore its normal activity after the treatment.

Thus, the effect could be not only glomerulus-specific, but rather nAChR-type-specific (in terms of subunit composition^19^) and cell-type-specific. Neuronal actors other than PNs and inhibitory LNs might also be involved. Excitatory cholinergic LNs have been described in the fruit fly, *Drosophila melanogaster*^30^,^31^,^32^, and there may be homologs in the honeybee. Moreover, the equivalent of a putative feedback neuron, ALF-1^33^, transmitting information from the mushroom bodies back to the ipsilateral antennal lobes, has been demonstrated in the fruit fly to be a cholinergic neuron^34^. Finally, cholinergic feedback onto the ORNs might also contribute to glomerulus-specific effects.

Another non-cholinergic effect that may contribute to imidacloprid’s interference with odour coding is its partial blocking of GABA-induced currents, observed for the first time in Kenyon cells^20^. All of these contributions might accumulate and give rise to the complex effects that we observed.

Our results add to evidences collected by others in conditioning studies showing that imidacloprid and other neonicotinoids interfere with olfactory learning^11^,^12^,^13^,^14^. We demonstrated here that malfunctioning of the olfactory pathway under neonicotinoid exposure starts as soon as at the AL level, where odour coding is disrupted. We suggest that imidacloprid impairs olfactory learning in several ways by acting at different locations in the bee brain. Along the odour processing pathway, odour discrimination is the first function to be impaired.

Finally, we would like to discuss an apparent contradiction between our results and those of Williamson and colleagues^12^, who in a classical conditioning study reported enhanced odour discrimination following acute treatment with imidacloprid (1.28 ng/bee). However, at higher doses^23^ or under chronic treatment^11^, imidacloprid impairs acquisition, memory, and discrimination. So, effects on the olfactory pathway are strictly dependent on doses. Our treatment is also acute, but instead of oral administration, the pesticide is bath-applied to the brain. Concentrations reaching the synapses are hard to compare but most likely higher in our case.

To conclude, we demonstrated for the first time an effect of a neonicotinoid pesticide, imidacloprid, on the AL functionality in the honeybee, *Apis mellifera*. The experimental result–that imidacloprid disrupts odour coding within the AL, reducing the EDs between odour pairs –, allows the inference of a decreased odour distinction capacity^35^. Diminished odour discrimination is likely to contribute to the previously reported impairment of olfactory learning and memory, since specific and robust stimulus encoding is necessary to form and recall odour-specific memory.

## Methods

### Animal preparation and staining

The procedure is adapted from Galizia and Vetter^36^. Forager honeybees were collected at the entrance of the beehive in a Plexiglass pyramid and fixed on a mounting stage after immobilisation at 4 °C. PNs were backfilled with the calcium indicator fura-2 in its dextran-conjugated form (ThermoFisher Scientific). A small volume of the crystallized dye was manually injected via a custom-made glass capillary at the intersection of the lateral- and medial-antenno-protocerebral tracts between medial and lateral calices of the MBs (for an image of the injection site see Paoli *et al*.^37^). To avoid lateral biases, left and right ALs were prepared alternately^38^,^39^. After injection, animals were fed on a 50/50 w/w sucrose solution and kept in the dark until imaging on the following day, allowing the dye to diffuse retrogradely into the AL.

### Data acquisition

Imaging was performed via a two-photon fluorescence microscope (Ultima IV, Bruker) combined with an ultra-short pulsed laser (Mai Tai Deep See HP, Spectra-Physics-Newport), tuned to 800 nm for fura-2 excitation^28^. The beam was focussed by a water-immersion objective (20x, NA 1.0, Olympus). The fluorescence was collected in epi-configuration, selected by a dichroic mirror, and filtered with a band-pass filter centred at 525 nm with 70 nm bandwidth (Chroma Technology Corp). Finally, it was detected by photomultiplier tube (Hamamatsu Photonics). An optimal signal-to-noise ratio was achieved with a laser power ≈10 mW, without any sign of photobleaching. The AL was repeatedly scanned by a set of galvanometric mirrors along a spiral line of interest crossing all glomeruli within a selected focal plane. The frame rate was ≈30 Hz. Changes in the intracellular calcium concentration manifested themselves as temporal variations of the fura-2 fluorescence intensity.

### Odour stimulation

Odour stimulation was performed through a custom-made olfactometer^40^, where a constant air stream is split into eight channels, each composed of two alternate paths: an odour chamber (1:500 dilutions in mineral oil) and a blank chamber (mineral oil). Single channels are switched by electronic valves controlled by a PCIe-6321 multifunction board (National Instruments) and programmed via a LabView-based user interface^41^. Acetophenone, benzaldehyde, 1-hexanol, 1-octanol (all Sigma-Aldrich) were applied sequentially as pulsed stimuli. Each odour pulse (duration: 1 s, inter-stimulus interval: 7 s) was repeated 25 times. Because the 4 odours were alternated, this produced an interval of 32 s between subsequent stimulations with the same odour.

### Imidacloprid administration

During the imaging sessions, the bee brain was continuously perfused via input and output capillaries, embedded laterally in the imaging mount. Via a peristaltic pump, Ringer’s solution (130 mM NaCl, 6 mM KCl, 4 mM MgCl_2_, 5 mM CaCl2, 160 mM sucrose, 25 mM glucose, 10 mM HEPES (all Sigma Aldrich), pH 6.7, 500 mOsm/L)^42^, was injected at a flow rate of ≈1 ml/min. During the treatment phase perfusion was switched for 60 s to Ringer’s with 10 μM imidacloprid (Sigma Aldrich) added to it.

### Data analysis

Images acquired via the microscope software Prairie View were de-noised and processed using custom-written MATLAB (Mathworks) codes. Glomerular response signals were extracted by manual identification of single glomeruli centres and averaged over 5-pixel intervals (1 μm/pixel) around those. The mean pre-stimulus activity *F* (averaged over 1 s) was subtracted and served as a normalization factor. The time series of this relative activity −Δ*F/F* were the basis for further analysis. The mean odour-response activity in each of the recorded glomeruli was obtained by averaging over a time window between 200 and 400 ms after stimulus onset. This period shows the maximal separation of PN odour response patterns in most bees. Glomeruli were identified as responsive if their average activity over the whole stimulus period deviated significantly from the background activity (*p* < 0.05: |Δ*F*|>*F* + *2σ*), and classified as either “excitatory” or “inhibitory” based on their sign. Response delays were calculated for significant responses, as the time from stimulus onset to time when half of the maximum activation was reached. To quantify odour separation in the *n*-dimensional glomerular space (*n* is the number of glomeruli recorded in each bee), Euclidean distances were calculated. The Euclidean distance *d*_*x,y*_ between two stimuli *x* and *y* is defined as the sum over all glomerular response differences:


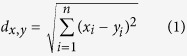


where *x*_*i*_ and *y*_*i*_ are the responses relative to odour *x* and odour *y* within glomerulus *i*, and *n* is the number of recorded glomeruli. To visualize the dynamics of the treatment effects on the odour response, dimensionality of that coding space was reduced by a principal component analysis (PCA). To be able to compare all bees within common coordinates, a transformation projected the whole coding space spanned by all glomerular responses (*n* = 172 (86 glomeruli pertaining to treated bees and 86 to control bees) × 4 odours) to principal components (PCs). The first 3 PCs explained all variance between the 172 × 4 glomerular responses averaged over the 4 pre-treatment stimuli (PC1: 47%, PC2: 36%, PC3: 17%). In the PC space, responses to each odour were averaged over the bees within the single groups (*n* = 5), producing a time sequence of 25 points corresponding to the 25 subsequent stimuli.

### Statistical analyses

- Figure 2b: the number of responding glomeruli and the average intensity of both excitatory and inhibitory responses were averaged across bees within each group (control bees: *n* = 5, and treated bees: *n* = 5). After assessing approximate normality of residuals and homoscedasticity of the samples (via Kolmogorov-Smirnov test and Levene’s test, respectively), in each case a two-way repeated measures ANOVA was performed (with time as within-subject factor and group as between-subject factor). Where ANOVA showed significant effects, pairs of values were further compared through paired (within one group) and unpaired (between groups) sample *t*-tests.
- Figure 3: EDs were calculated relative to the six odour pairs and the three time windows in each bee. ED changes were normalized with respect to the pre-treatment values: (ED_after_ − ED_before_)/ED_before_, applied to both time points 1 min and 8 min after end of treatment (EOT). We then averaged across odour pairs (*n* = 6) and bees per group (*n* = 5). Results were compared via a two-way repeated measures ANOVA (after assessing normality of residuals and homoscedasticity of the samples), with time as repeated measure and group as between-subject factor.
- Figure S1: the first 24 repetitions of 1-octanol stimulation in a treated bee were averaged in groups of 4 to obtain the six subsequent windows shown in this figure. One-way ANOVA (after assessing normality of residuals and homoscedasticity of the samples) was performed to compare values of peak response (the maximum after stimulus onset) and the integral of the response (area under the curve of response from stimulus onset to return to baseline) in these six “groups”. Via Dunnett’s *post-hoc* test, all later time windows were tested against the first.
- Figure S4: glomeruli that showed significant odour-evoked responses both before treatment and after EOT–either at 1 min after EOT or 8 min after EOT–were used for comparisons. Differences in onset delays were extracted from pairs of significant responses and compared via two-way ANOVA, with time and group as two between-subject factors. Two-way ANOVA was chosen because we wanted to test a possible effect of interaction of these two factors. However, since the analysis showed no significant effect of time × group, we further compared the two groups via a Kruskal-Wallis test, more suitable in this case, as residuals deviated from normality.

## Additional Information

**How to cite this article**: Andrione, M. *et al*. Neonicotinoid-induced impairment of odour coding in the honeybee. *Sci. Rep.* **6**, 38110; doi: 10.1038/srep38110 (2016).

**Publisher's note:** Springer Nature remains neutral with regard to jurisdictional claims in published maps and institutional affiliations.

## Supplementary Material

Supplementary Material
