# Supplementary material for: Neonicotinoid-induced impairment of odour coding in the honeybee
Source: Sci Rep. 2016 Dec 1;6:38110. doi: 10.1038/srep38110 (PMC5131477; doi:10.1038/srep38110)
Supplement: Supplementary Material [file srep38110-s1.pdf]

Neonicotinoid-induced impairment of odour coding in the honeybee.

Mara Andrione[1 *], Giorgio Vallortigara[1], Renzo Antolini[2,1], and Albrecht Haase[2,1 *]

1 Center for Mind/Brain Sciences, University of Trento, Rovereto, Italy

2 Department of Physics, University of Trento, Trento, Italy

Supplementary Figure S1

Odour-evoked activity in a single glomerulus of a treated bee.

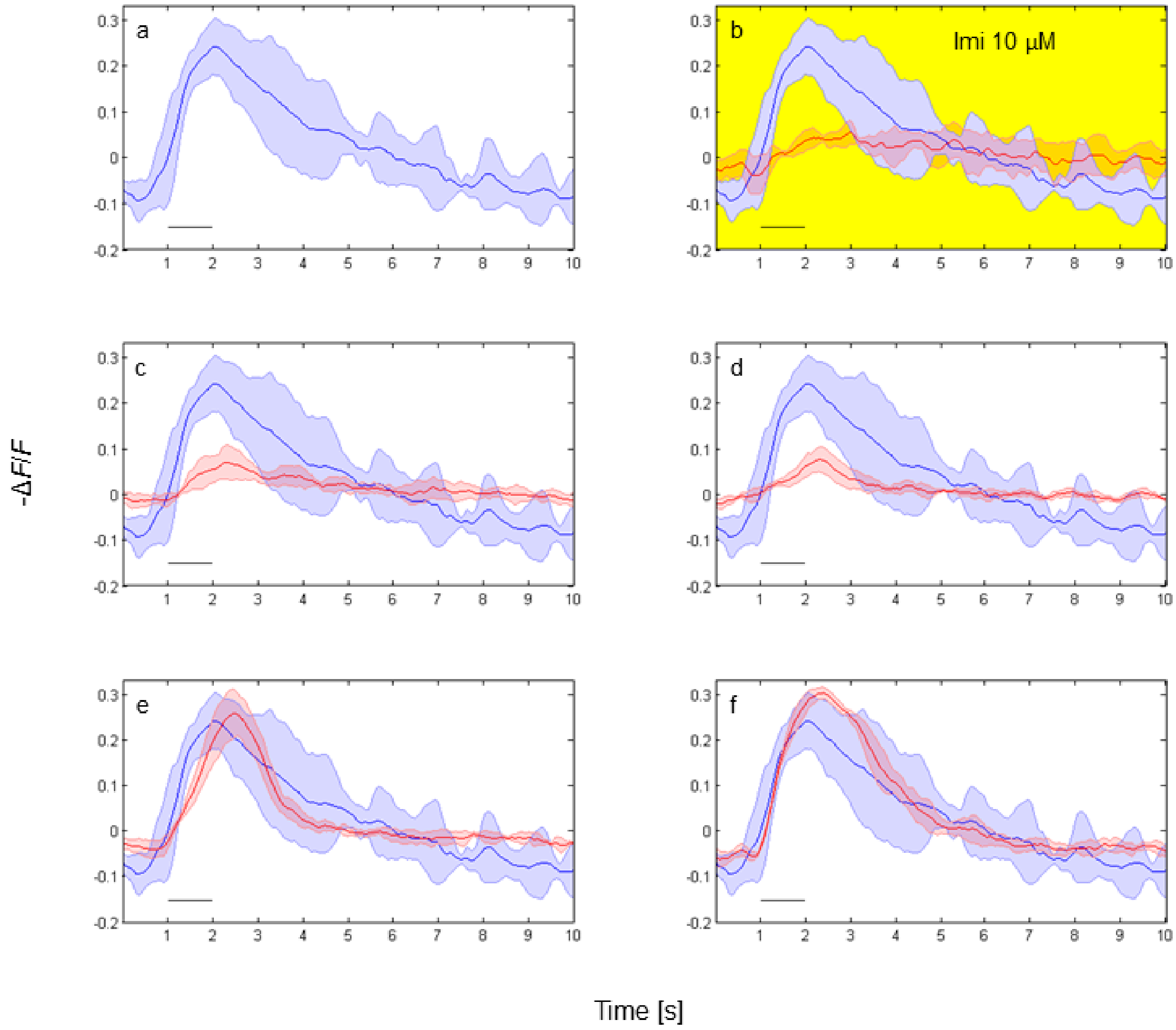

Supplementary Figure S1. Odour-evoked activity in a single glomerulus, identified as GloT1-17, in a treated bee. Boxes (a-f) represent subsequent time windows. In each, the change in fluorescence over time (blue in a, red in b-f) is averaged across four subsequent repetitions of the odour stimulus (1-octanol; horizontal black line). In (b-d) both peak response (one-way ANOVA, $F_{5,18}=34$, $p<10^{-7}$, followed by Dunnett's *post hoc* test) and integral response (one-way ANOVA, $F_{5,18}=16$, $p<10^{-5}$, followed by Dunnett's *post hoc* test) are reduced with respect to (a). Shadows represent standard deviations. The profile of response in (a) is reported (in blue) in all the subsequent windows for comparison. In yellow: the time window (b) corresponding to imidacloprid application. Before and after: physiological (Ringer's solution) washing.

Supplementary Figure S2

Odour-evoked activity in a single glomerulus before and after the application of a 1 µM imidacloprid solution.

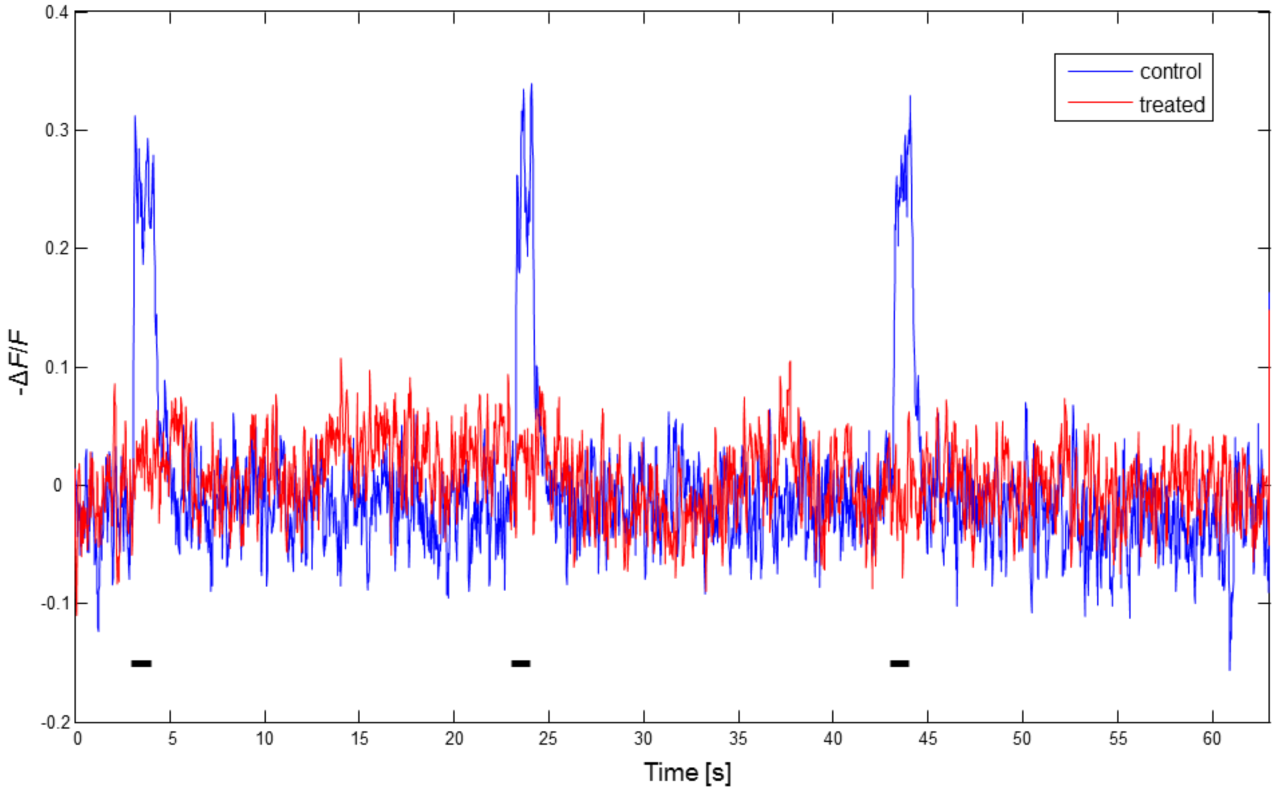

Supplementary Figure S2. Odour-evoked activity (odour pulses are represented by the black horizontal bars) in a single glomerulus T1-37 before (blue) and after (red) the application of a 1 µM imidacloprid solution. Odour of stimulation is benzaldehyde. Imidacloprid solution is applied in a time-unrestricted manner, in substitution of the whole volume of haemolymph covering the brain surface. In this setting, there is no movement of the buffer solution or washing phase. 2-3 min after the application of a concentration of the drug as low as 1 µM, the odour-evoked activity is lost. In the perfusion set-up, used in later experiments, effects comparable to those that are shown here set in only at a concentration 10 times higher.

Supplementary Figure S3

Odour-evoked activity and odour-specific recovery in a single glomerulus.

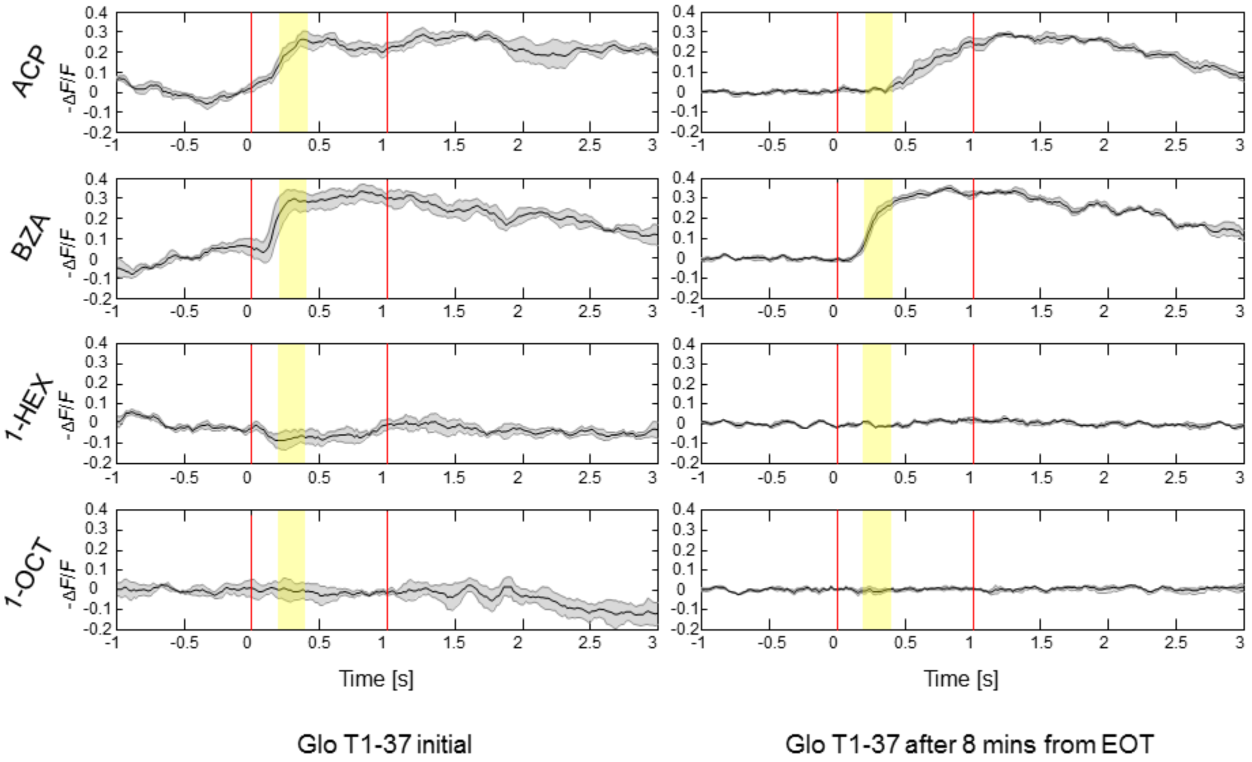

Supplementary Figure S3. Odour-evoked activity and odour-specific recovery in a single glomerulus, identified as GloT1-37, in a treated bee, from 1 s prior to stimulus onset to 2 s after stimulus offset. The glomerular activity is shown in response to the four different odours used in the stimulation paradigm (ACP=acetophenone, BZA=benzaldehyde, HEX= 1-hexanol, OCT= 1-octanol). Each window represents the average -Δ*F/F* calculated over the course of three subsequent repetitions before (left column) and 8 min after the EOT (right column). While the recovery of the response to BZA is complete, the recovered response to ACP is delayed (compared to the beginning), such that it will not show up in the peak activity calculated at 200-400 ms (yellow area) after stimulus onset (see Fig. 1 for comparison). Red vertical bars: stimulus onset and offset. Grey shadows represent SEM.

Supplementary Figure S4

Change in response delay.

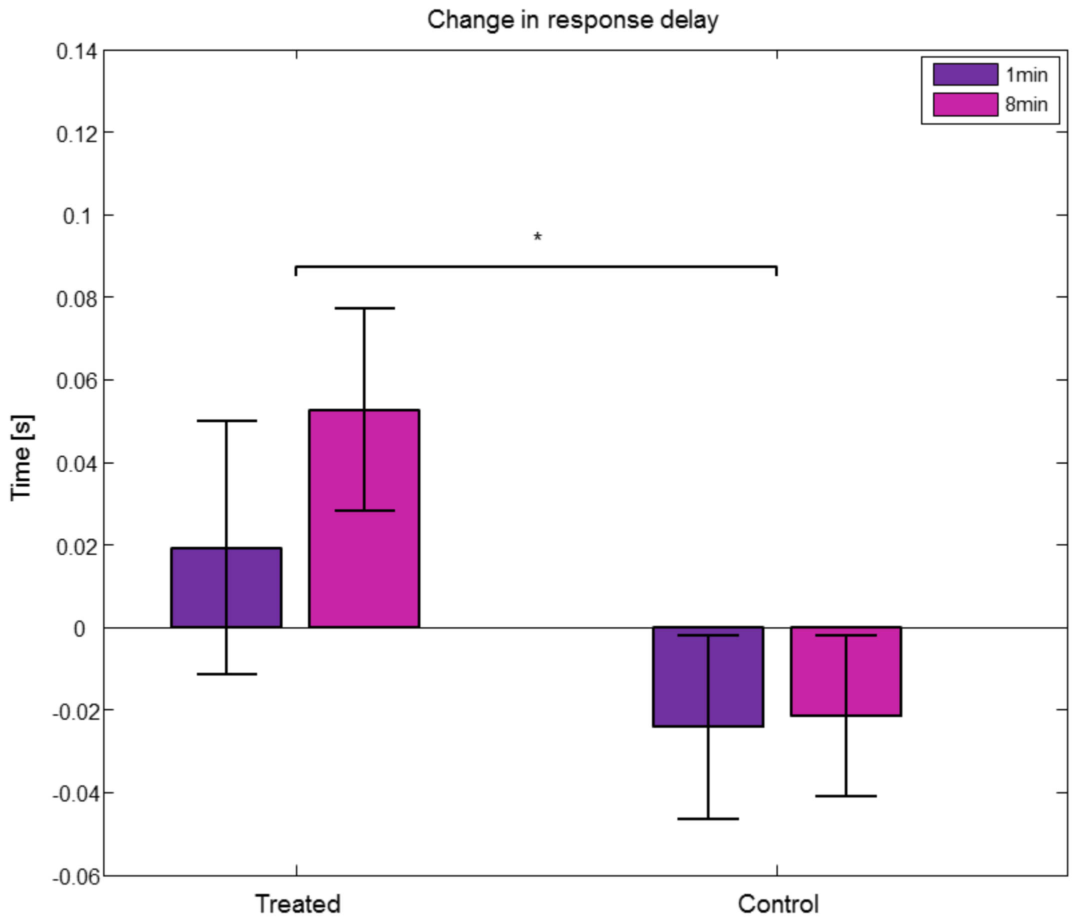

Supplementary Figure S4. Change in delay of the response onset $delay_{after}$-$delay_{before}$ at 1 min (in violet) and 8 min (in fuchsia) after EOT. Only active glomeruli were considered (*i.e.* those in which the mean -Δ*F/F* during stimulus pulse deviated from the mean -Δ*F/F* calculated during 1 s before stimulus by at least 2 standard deviations; *n*=76, 88, 138, 135 odour responses). Error bars represent SEM. The two groups show significantly different delays in response onset (Kruskal-Wallis, H_1=6.2, *p*=0.013).

Supplementary Figure S5

Euclidean distances between pairs of odours in a treated and a control bee.

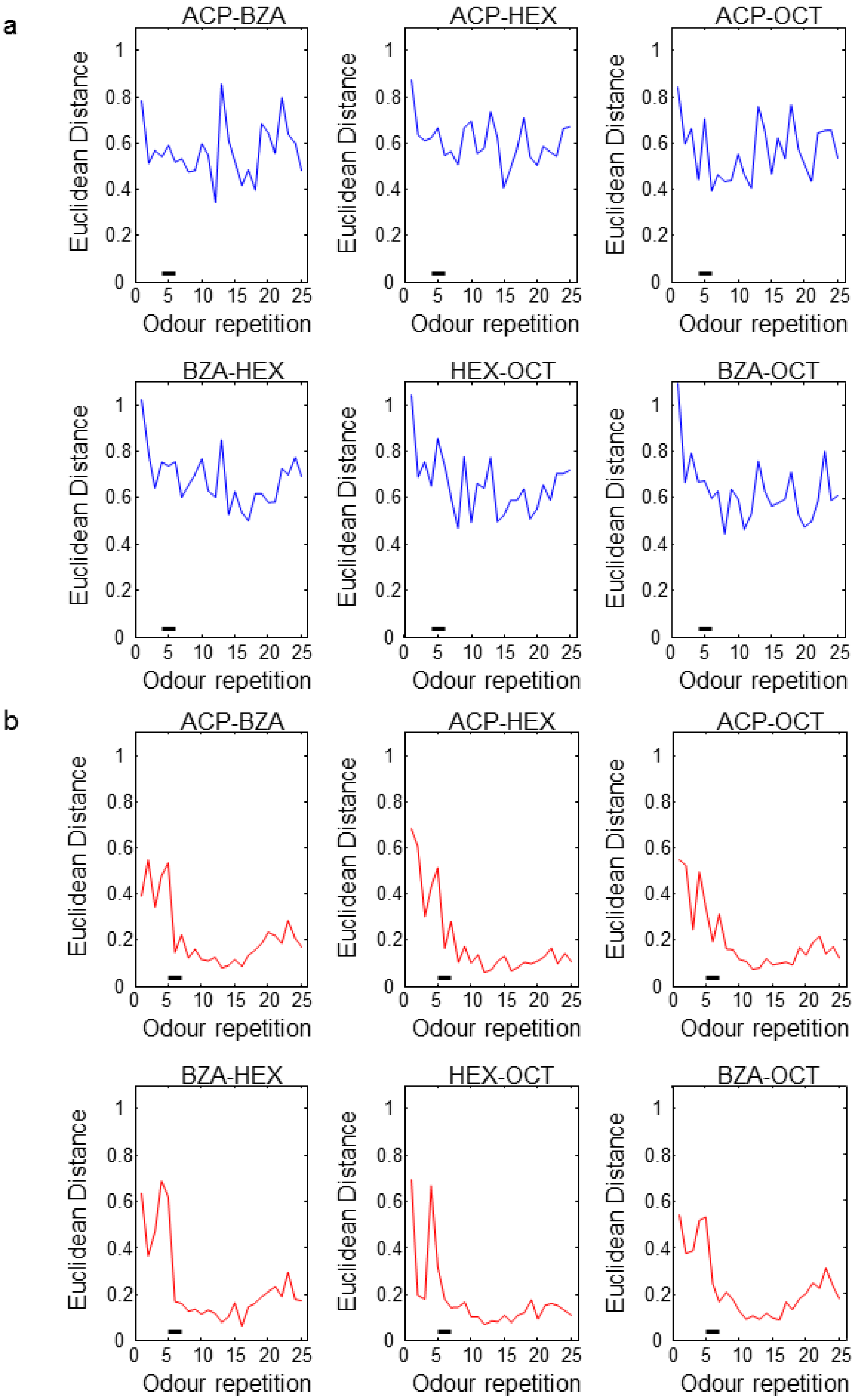

Supplementary Figure S5. Euclidean distances between pairs of odours (ACP= acetophenone, BZA= benzaldehyde, HEX= 1-hexanol, OCT= 1-octanol) across 25 stimulations within one control (a) and one treated (b) bee. The EDs are strongly reduced in the treated bee by the treatment (black horizontal line) with a slight tendency for recovery in the later repetitions.

Supplementary Figure S6

Euclidean distances between pairs of odours in control and treated bees.

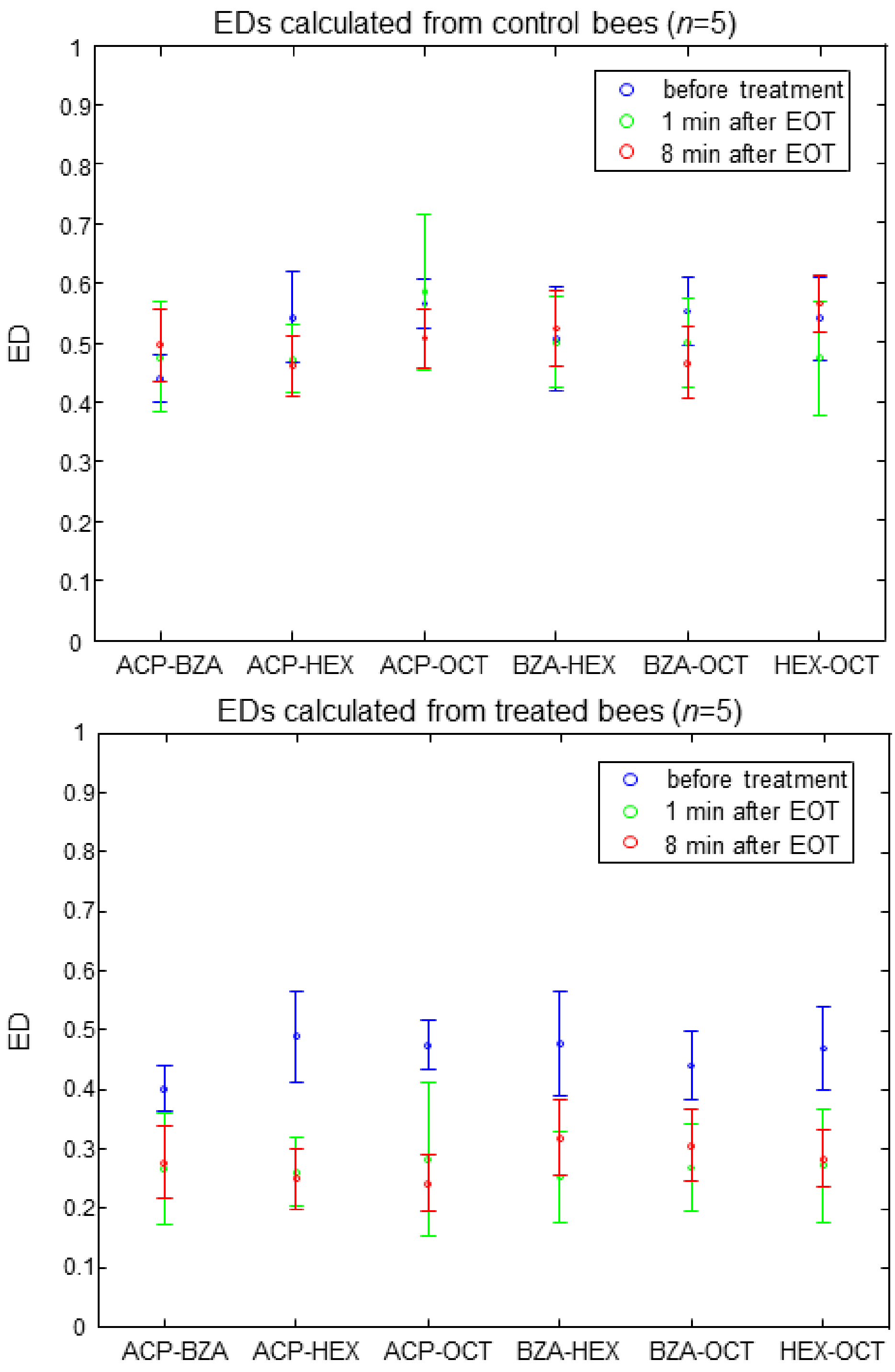

Supplementary Figure S6. Average Euclidean distances between pairs of odours calculated before the treatment (blue), at 1 min (green), and 8 min (red) after EOT, in controls (above) and treated bees (below). Error bars show SEM (*n*=5 bees per group).
